# Arthroscopic cuff repair: footprint remnant preserving versus debriding rotator cuff repair of transtendinous rotator cuff tears with remnant cuff

**DOI:** 10.1186/s12891-024-07431-z

**Published:** 2024-04-17

**Authors:** Jae Min Lee, Jong-Hun Ji, Sang-Eun Park, Dongwhan Suh, Ki-Jeon Song

**Affiliations:** 1 Department of Orthopedic Surgery, Shinsegae Seoul Hospital, Seoul, Korea; 2 Department of Orthopedic surgery, College of Medicine, The Catholic University of Korea, Seoul, Republic of Korea; 3 Department of Orthopedic surgery, Daejeon st. mary’s hospital. College of Medicine, The Catholic University of Korea, 64, Daeheung-ro, Jung-gu, Daejeon, 34943 South Korea

**Keywords:** Transtendinous rotator cuff tears, Remnant cuff, Remnant preserving rotator cuff repair, Tendon to tendon healing, Repaired tendon quality

## Abstract

**Background:**

In transtendinous full thickness rotator cuff tears (FTRCT) with remnant cuff, conventionally, cuff remnant of the greater tuberosity (GT) is debrided for better tendon to bone healing. However, larger cuff defect caused overtension on the repaired tendon. The purpose of this study was to compare the clinical outcomes and tendon integrity between remnant preserving and remnant debriding cuff repairs in the transtendinous FTRCT with remnant cuff.

**Methods:**

From March, 2012 to October, 2017, a total of 127 patients who had the transtendinous FTRCT with remnant cuff were enrolled in this study. Rotator cuff tears were repaired arthroscopically, with patients divided into two groups: group I (*n* = 63), where rotator cuff remnants were preserved during the repair, and group II (*n* = 64), where the remnants were debrided during the repair. Clinical outcomes were assessed at the last follow-up (minimum 2 years) using the UCLA score, ASES score, SST score, Constant Shoulder score, and range of motion (ROM). The analysis of structural integrity and tendon quality was performed using the Sugaya classification on postoperative MRI scans at 8 months after surgery.

**Results:**

At the final follow-up, UCLA, ASES, SST, and CS scores significantly improved from preoperative values to postoperative (all *p* < 0.05): UCLA (I: 19.6 ± 6.0 to 31.7 ± 3.2, II: 18.0 ± 5.7 to 31.5 ± 3.2), ASES (I: 54.3 ± 10.7 to 86.5 ± 12.5, II: 18.0 ± 5.7 to 85.8 ± 12.4), SST (I: 5.6 ± 2.8 to 10.2 ± 2.0, II: 5.0 ± 2.9 to 10.1 ± 2.5), CS (I: 74.0 ± 17.2 to 87.8 ± 9.7, II: 62.0 ± 19.2 to 88.3 ± 6.2). However, there were no significant differences between the two groups (*p* > 0.05). Also, remnant preserving cuff repair yielded significantly better tendon quality on postoperative MRI (*p* < 0.05). The incidence of re-tear (Sugaya’s Type IV and V) was not significantly different between the two groups (I:17% vs. II:19%; *p* = 0.053).

**Conclusions:**

Remnant preserving rotator cuff repairs, which facilitate tendon-to-tendon healing, are superior in terms of tendon quality and are the preferred option for transtendinous FTRCT.

**Trial registration:**

Retrospectively registered.

**Supplementary Information:**

The online version contains supplementary material available at 10.1186/s12891-024-07431-z.

## Background

Rotator cuff repairs are performed for the purpose of functional recovery and pain relief of the disabled shoulder joint. However, even in cases where rotator cuff tears are completely repaired, patients may present with dissatisfaction and functional disability. Usually, during the rotator cuff repair, remnant of the rotator cuff tendon insertion on the greater tuberosity (GT) is removed or debrided completely and the GT is decorticated for better tendon to bone healing. In addition, re-establishment of rotator cuff footprint during rotator cuff repair is considered crucial to improve initial rotator cuff repair strength and tendon to bone healing [1]. During the rotator cuff repair, a medially retracted tendon is pulled up to GT for covering the footprint as part of the effort of original anatomical footprint restoration. However, after complete debridement of remnant rotator cuff on the GT in the full thickness rotator cuff tear (FTRCT), larger defect between GT and retracted tendon was developed and an anatomical rotator cuff repair necessitated greater tension on the retracted tendon. In addition, rotator cuff repair with complete debridement of remnant rotator cuff means that the normal transitional zone does not remain in the tendon-to-bone interface [2]. As a result, after rotator cuff repair, the subsequent tendon-to-bone healing will be weak due to the physical property differences. Furthermore, pulled repaired tendon could give rise to tension overload and re-tear of the repaired rotator cuff tendon [3]. In these type tears with remnant rotator cuff, sometimes, a robust and sufficient footprint of tendon remnants on the GT can be observed. This is a relatively rarely described tear pattern and is similar to previously reported traumatic transtendinous FTRCT in young athletes [4]. Also, this configuration is similar to the type 2 re-tear pattern [5] or L-shape tear pattern with infraspinatus tendon remnant which Mochizuki et al. [6] described as new humeral insertion of the supraspinatus and infraspinatus tendon on the GT. In such tear pattern, efforts should be directed towards preserving a robust and sufficiently thick remnant tendon on GT to reduce tension overload on the repaired tendon and also to provide tendon to tendon healing, rather than tendon to bone healing. In this study, we conducted a case-control study to compare rotator cuff remnant preserving repair with remnant debriding repair for transtendinous FTRCT. We evaluated clinical outcomes and assessed the integrity and quality of the repaired tendon using postoperative MRI as outcome measures.

The purpose of this study was to compare the clinical outcomes and tendon integrity between rotator cuff remnant preserving and remnant debriding rotator cuff repairs in the transtendinous FTRCT with remnant cuff. Our hypothesis was that remnant preserving rotator cuff repair in the transtendinous FTRCT with remnant cuff have better clinical outcomes and better tendon integrity of repaired tendon than remnant debriding rotator cuff repair.

## Materials and methods

### Patient enrollment

**Fig. 1:**
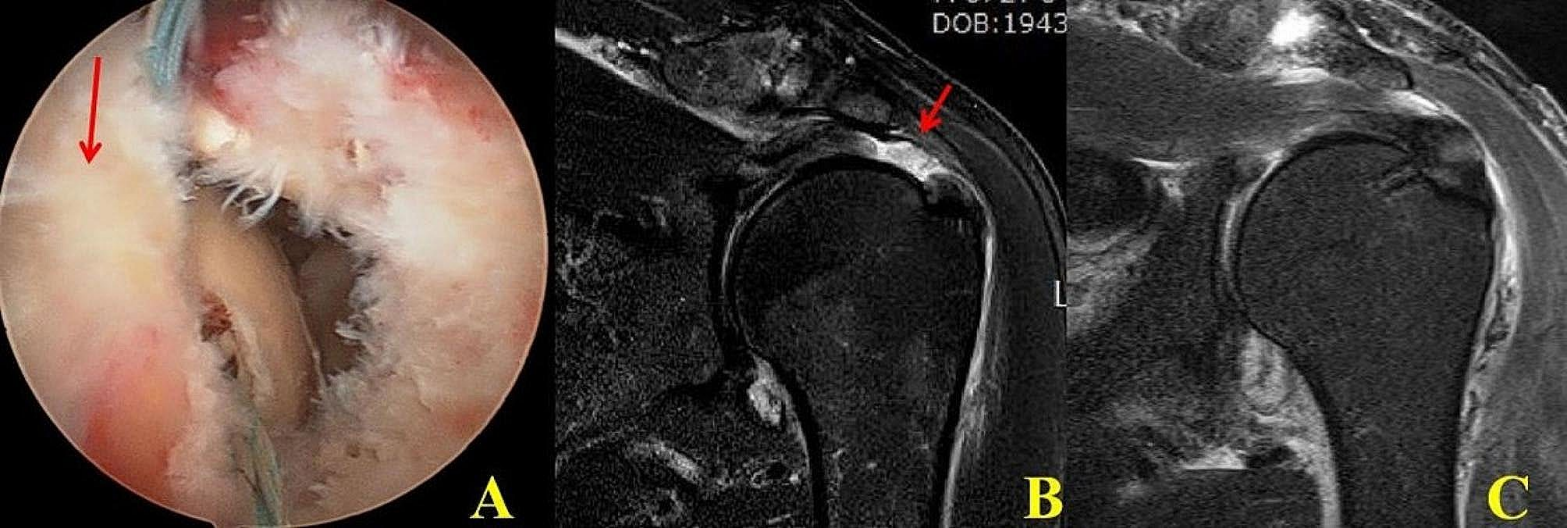
Classical transtendinous with remnant cuff tendon. (**A**) This classic type IIA configuration showed remnant rotator cuff was present on whole anteroposterior length of the greater tuberosity footprint. (**B**) On the preoperative and postoperative coronal MRI, transtendinous full thickness rotator cuff tear with 1 cm sized remnant cuff and tendon defect (arrow) was shown. (**C**) Postoperative follow-up MRI showed sufficient thickness well healed (good quality) repaired tendon

This retrospective comparative study was ethically approved by the institution’s ethics committee (DC17RESI0049) and informed consent was obtained from all the participants. From March, 2012 to October, 2017, total 127 patients with the transtendinous FTRCT with remnant cuff underwent arthroscopic repair and were enrolled in the study. The remnant cuff was defined as the presence of minimum 10 mm of anteroposterior and mediolateral supraspinatus remnant on the rotator cuff insertion site (GT), a similar width as the original footprint of supraspinatus [4, 7, 8]. The tear pattern was suspected by preoperative MRI and confirmed with arthroscopy at the time of repair (Fig. 1). The inclusion criteria were: (1) patients with FTRCT on MRI, (2) availability for more than 2-year follow-up, and (3) about 10 mm of mediolateral remnant as measured on arthroscopy. Exclusion criteria were: (1) advanced degenerative and small atrophic remnant cuff < 10 mm on arthroscopy, (2) massive rotator cuff tears i.e. associated full thickness subscapularis and infraspinatus tears with retraction (3) revision surgery, and (4) osteoarthritis. (5) partial thickness rotator cuff tears. In our study, 4 patients which were suspected to have transtendinous FTRCT on preoperative MRI were found to have grade 3 bursal tear with remnant and were not included in the study.

### Evaluation of clinical outcomes and ROM

Clinical evaluation was done preoperatively and compared with the last follow-up visit evaluation (mean, 24 ± 6 months; range, 18–30 months). The University of California at Los Angeles (UCLA) score, American Shoulder and Elbow Surgeons (ASES) score, Simple Shoulder Test (SST) score, Constant Shoulder (CS) score, and ROM were included in clinical evaluation.

### Evaluation of preoperative and postoperative rotator cuff on MRI

**Fig. 2:**
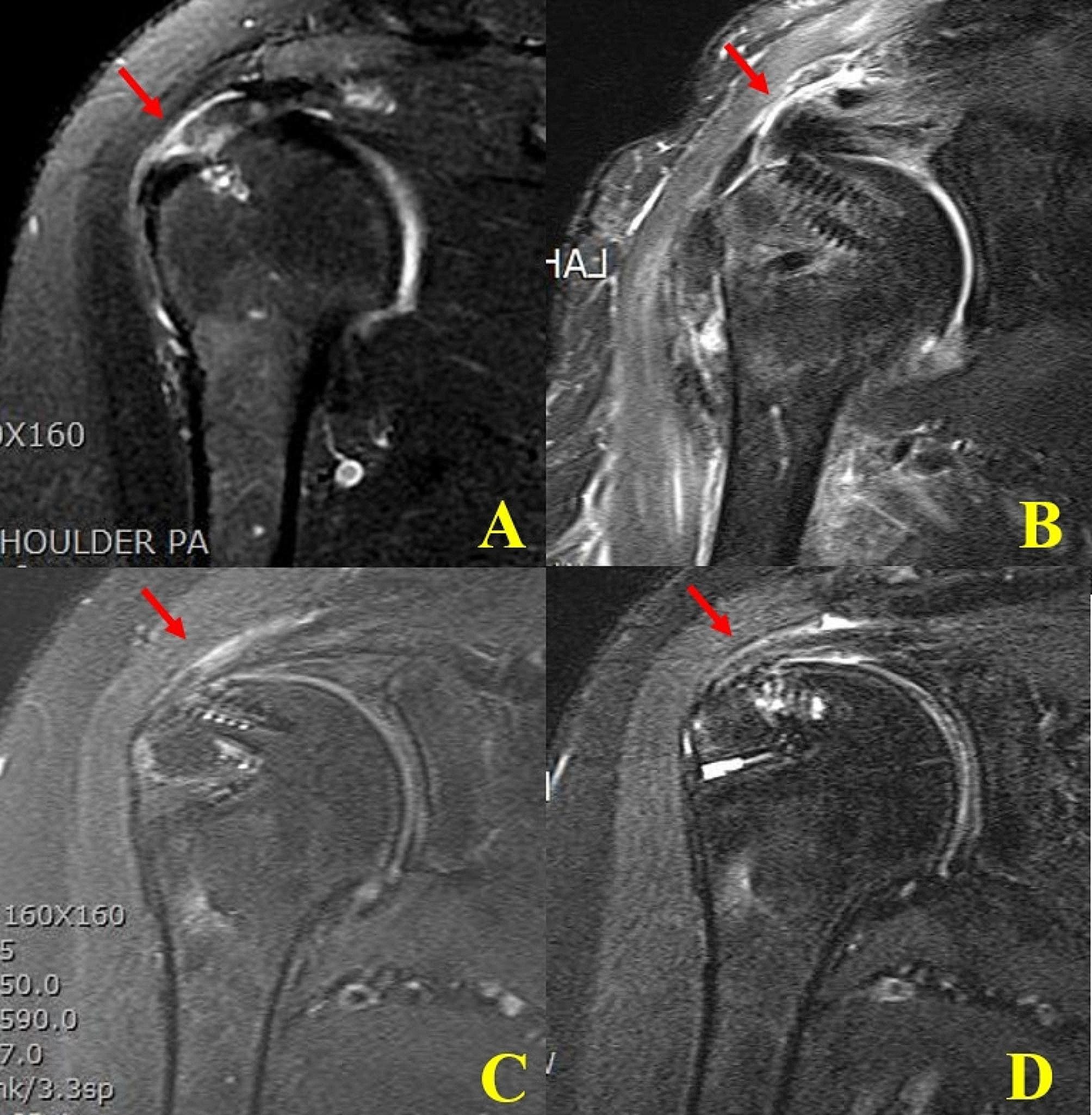
The preoperative and postoperative MRI scans. (**A**) The oblique coronal FSE T2-weighted image clearly showed the presence of the footprint remnant tendon in the transtendinous full thickness rotator cuff tears (arrow). (**B**), (**C**), (**D**) Immediate, postoperative 1, and postoperative 6 months follow-up MRI showed intact cuff integrity without high signal intensity (arrow)

The tear size, degree of tendon retraction, presence of remnant tendon of the GT footprint and the degree of muscle fatty infiltration were measured on preoperative MRI scans. The remnant tendon on the GT footprint was evaluated on the oblique coronal FSE T2-weighted images. Re-tear after rotator cuff repair usually occurs between 3 and 6 months after surgery [9, 10]. Thus, postoperative follow-up MRI scan was done 8 months after surgery and tendon integrity was evaluated (Fig. 2). The repaired tendon was classified into five categories as suggested by Sugaya et al. [11] We categorized patients who underwent rotator cuff repair into two groups based on the tissue quality of the repaired tendon. Sugaya classification Types I and II, labeled as “No tear” were seen as good quality tendons. On the other hand, Types III, IV, and V, indicating partial tear (Type III) and full thickness tear (Type IV and V), were considered poor quality tendons due to the presence of tears [12]. All the MRI evaluations were done by clinical fellows at our institute who were blinded to the operative technique of repair.

### Surgical procedure (Fig. 3)

**Fig. 3:**
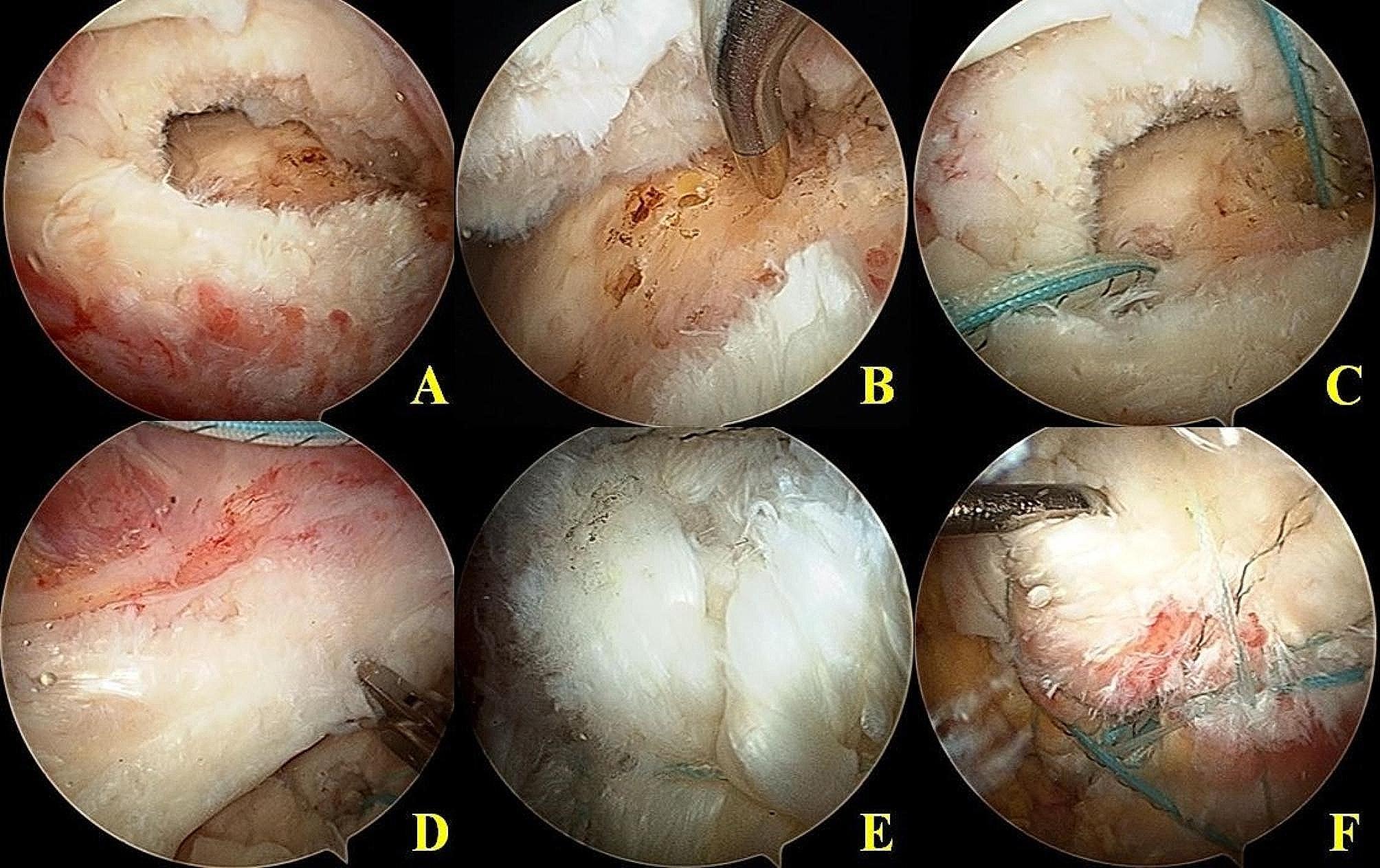
Surgical technique of remnant preserving suture bridge rotator cuff repair in the transtendinous full thickness rotator cuff tears. (**A**) After debridement of the subacromial bursa, torn rotator cuff with remnant cuff was found. (**B**) Minimal decortication and multiple channeling of the greater tuberosity bone bed was performed. (**C**) Medial row anchor was inserted through the Neviaser portal. (**D**) In medial row repair, the repair site should avoid the musculotendinous junction. Only small tendinous portion was found in this transtendinous full thickness rotator cuff tears. The medial row sutures were passed through the tendinous portion medial to the musculotendinous junction. (**E**) After remnant preserving rotator cuff repair, all greater tuberosity was covered by the repaired rotator cuff. (**F**) Probe was inserted into the gap between remnant cuff and repaired cuff

**Fig. 4:**
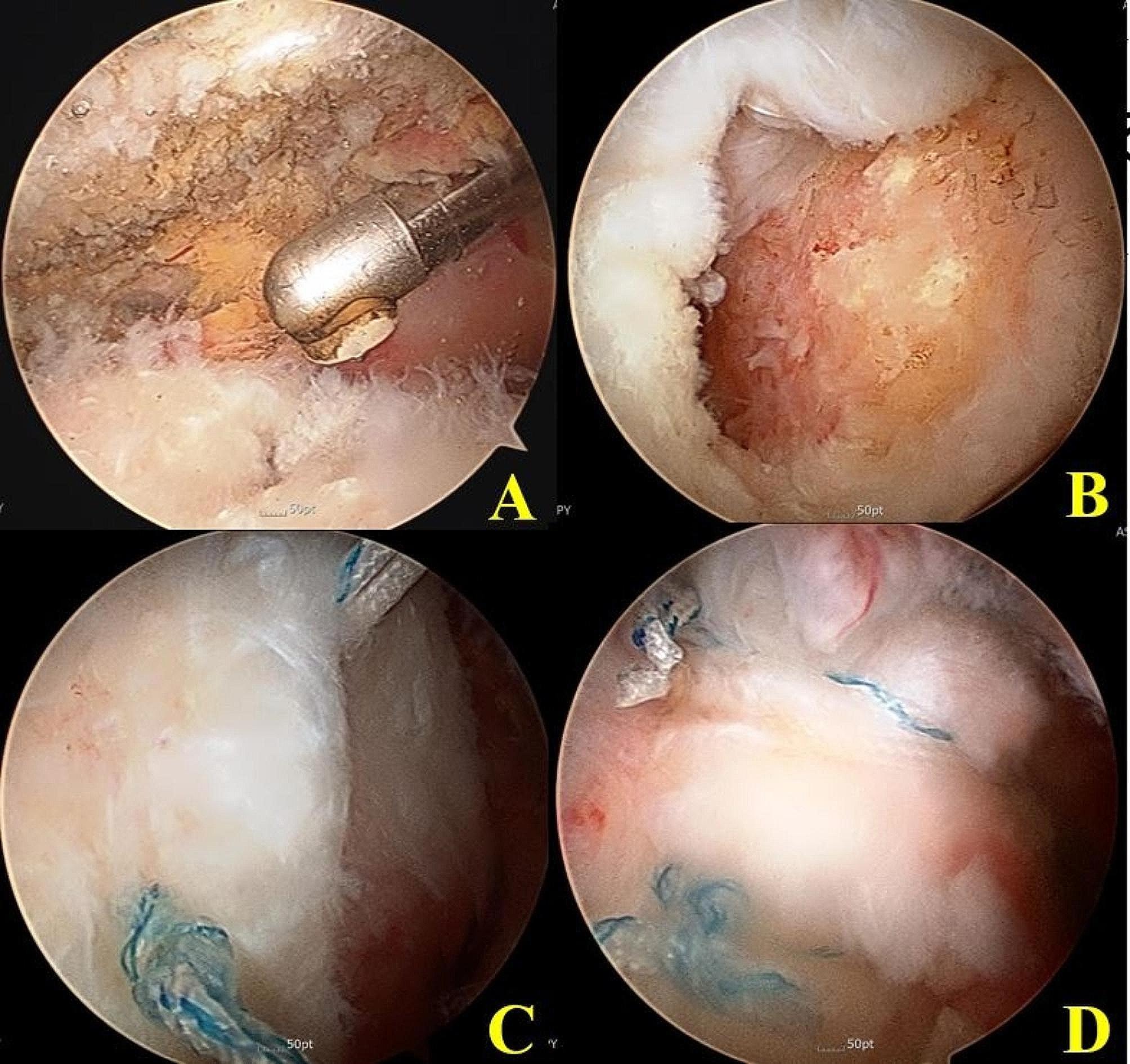
Arthroscopic view in the remnant debriding rotator cuff repair. (**A**) The remnant cuff was present on the footprint of greater tuberosity. (**B**) All remnant cuff was complete debrided. (**C**), (**D**) Conventional suture bridge rotator cuff repair was performed

All operative procedures were performed by the senior author of the study with the patient in the lateral decubitus position under general anesthesia supplemented with interscalene block. Standard posterior viewing and anterior working portals were used for diagnostic examinations and to address any intra-articular lesions. After the standard examination of the glenohumeral joint and treatment of intra-articular injuries, the arthroscope was moved to the subacromial space. Bursectomy and subacromial decompression were minimally performed until a clear view of the rotator cuff was obtained. After debridement of the torn tendon margin, tear configuration of the remnant tendon was evaluated (Fig. 3A). After evaluation of tear size, tear configuration, and tendon mobility, the size of the footprint remnant tendon (anteroposterior length and mediolateral width) was checked using a calibrated probe. For preservation of cuff remnants on the GT footprint, we minimized debridement of cuff remnants and decortication of the GT footprint. Medial row anchors were inserted by the Neviaser portal to get 90º insertion angle in most of the remnant preserving repairs because due to the coverage of humeral head almost up to the articular margin by remnant cuff, appropriate trajectory for anchor insertion could not be achieved with lateral portal. Without complete removing remnant cuff, the site of anchor insertion was just medial to the footprint remnant and near the lateral articular cartilage margin of the humeral head. Through multiple channeling on the tuberosity, extrusion of marrow fat globules was confirmed (Fig. 3B). If anchors were inserted at deadman angle (classical 45º anchor insertion) from lateral portal, a remnant cuff on the GT would interfere with anchor insertion and it would not be possible to insert anchors without sacrificing the remaining remnant cuff. Medial row anchor insertion through the Neviaser portal is a very useful technical tool for remnant cuff preservation (Fig. 3C). Another difficulty a more proactive approach to surgical intervention in these transtendinous tears is smaller tendinous portion of torn cuff than the conventional rotator cuff tears. So, only a very small portion of tendinous tissue is available for suture passage. (Fig. 3D). After medial row repair, conventional lateral row repair was performed (suture bridge repair). All suture limbs of the medial row anchor were passed using a retrograde shuttle relay technique with a suture hook with a crescent shape through the retracted torn cuff and medial row repair was performed. These medial row repair approximated and interdigitated both ends of the tendon to increase the contact area for tendon-to-tendon healing. Then, the lateral row anchors were inserted at just lateral to the GT (An additional movie file shows this in more detail [see Additional file. 1], available at https://bmcmusculoskeletdisord.biomedcentral.com; Fig. 3E, F). Meanwhile, in remnant debriding rotator cuff repair, we performed the same suture bridge rotator cuff repair technique, which involves complete debridement of all remnant tissue on the tuberosity. (Fig. 4). During the initial phase of our study, we favored remnant debriding cuff repair due to its facilitation of easier anchor insertion and a quicker surgical procedure compared to the technique of preserving the remnant cuff. Nevertheless, due to advancements in remnant cuff management techniques, including focal debridement of remnant cuffs, minimal GT decortication with multiple channelling, and the ability to perform adequate anchor insertion through the Neviaser portals, we transitioned to performing remnant preserving cuff repairs during the later phase of our study.

### Statistical analyses

A *p* value < 0.05 was defined as statistically significant. All statistical analyses were performed using the SPSS statistical package version 19.0 (SPSS Inc, Chicago, Illinois). Measurements were expressed as mean ± standard deviation with confidence interval 95% for continuous variables. To compare both groups, we used the nonparametric test method, Mann-Whitney U, because the normality test showed no normality. Cross-validation (Chi- square) was used to determine if there were differences in patient characteristics according to the groups. Fisher’s validation was performed for accurate verification. Wilcoxon test, a nonparametric test method, was used for comparison between preoperative and postoperative scores. On postoperative follow-up, crossover analysis (Chi square) was used for comparison between the two groups for repaired tendon quality and re-tear. Fisher’s validation was performed for accurate verification. In the power analysis, the power was estimated utilizing effect size 0.5, alpha error accepted was 0.05, and the beta error was 0.2. The study had a power of 86%.

## Results

### Baseline demographics

We divided the 127 patients (mean age, 60 ± 9 years; range, 37–76 years) with transtendinous FTRCT with remnant cuff who underwent rotator cuff repair into footprint remnant preserving repair group (group I, *n* = 63) and remnant debriding group (group II, *n* = 64). We evaluated and compared age, sex ratio, trauma history, pain duration at the time of the hospital visit, and concomitant procedures between the two group. The distribution of age, sex, body mass index, trauma history, pain duration, and concomitant procedures were not significantly different between the two groups (all *p* > 0.05) (Table 1).

**Table 1:** Patient Demographic Data

	Group I (Remnant preserving) (*n* = 63)	Group II (Remnant debriding) (*n* = 64)	*p* value
Age at surgery, yr	62.0 ± 9.3	59.7 ± 9.1	0.173
Sex ratio, male: female, n	26:37	31:33	0.791
Trauma history, n	13	15	0.145
Pain duration, mo	10.0 ± 13.9	11.1 ± 15.3	0.306
Concomitant procedure, n			
Acromioplasty	33	31	0.125
Biceps tenotomy & tenodesis	10	15	0.243
Subscapularis repair	3	6	0.076

NOTE. Data are presented as mean ± standard deviation unless otherwise indicated.

### Evaluation of clinical outcomes and ROM

In both groups, clinical scores and ROM improved significantly at final follow-up period as compared to preoperative status (all *p* < 0.05). However, there was no statistically significant difference between the two groups in terms of clinical scores and ROM (all *p* > 0.05) (Table 2) (Table 3).

**Table 2:** Comparison of Clinical Outcomes Between Two Groups In Preoperative and Postoperative Follow Up

	Group I (Remnant preserving) (*n* = 63)	Group II (Remnant debriding) (*n* = 64)	*p* value
UCLA			
Preoperative	19.6 ± 6.0	18.0 ± 5.7	0.285
Postoperative 2yr	31.7 ± 3.2	31.5 ± 3.2	0.689
*p* value	< 0.001	< 0.001	
ASES			
Preoperative	54.3 ± 10.7	18.0 ± 5.7	0.170
Postoperative 2yr	86.5 ± 12.5	85.8 ± 12.4	0.801
*p* value	< 0.001	< 0.001	
SST			
Preoperative	5.6 ± 2.8	5.0 ± 2.9	0.485
Postoperative 2yr	10.2 ± 2.0	10.1 ± 2.5	0.893
*p* value	< 0.001	< 0.001	
CS			
Preoperative	74.0 ± 17.2	62.0 ± 19.2	0.152
Postoperative 2yr	87.8 ± 9.7	88.3 ± 6.2	0.890
*p* value	< 0.001	< 0.001	

NOTE. Data are presented as mean ± standard deviation.

UCLA, The University of California at Los Angeles; ASES, American Shoulder and Elbow Surgeons; SST, Simple Shoulder Test; CS, Constant Shoulder.

**Table 3:** Comparison of Range Of Motion Between Two Groups In Preoperative and Postoperative Follow Up

	Group I (Remnant preserving) (*n* = 63)	Group II (Remnant debriding) (*n* = 64)	*p* value
Flexion			
Preoperative, °	143.0 ± 22.7	133.3 ± 36.0	0.596
Postoperative 2 year, °	154.1 ± 12.5	153.2 ± 17.1	0.755
*p* value	< 0.001	< 0.001	
Abduction			
Preoperative, °	136.0 ± 26.2	137.0 ± 91.2	0.637
Postoperative 2 year, °	153.6 ± 13.4	152.0 ± 18.6	0.624
*p* value	< 0.001	< 0.001	
External rotation			
Preoperative, °	25.4 ± 15.7	26.0 ± 18.2	0.859
Postoperative 2 year, °	28.8 ± 17.5	30.6 ± 20.1	0.632
*p* value	< 0.001	< 0.001	
Internal rotation			
Preoperative, °	33.0 ± 12.0	26.0 ± 11.4	0.062
Postoperative 2 year, °	40.7 ± 13.5	34.0 ± 15.1	0.081
*p* value	0.005	0.004	

NOTE. Data are presented as mean ± standard deviation unless otherwise indicated.

### Evaluation of preoperative and postoperative rotator cuff on MRI

**Fig. 5:**
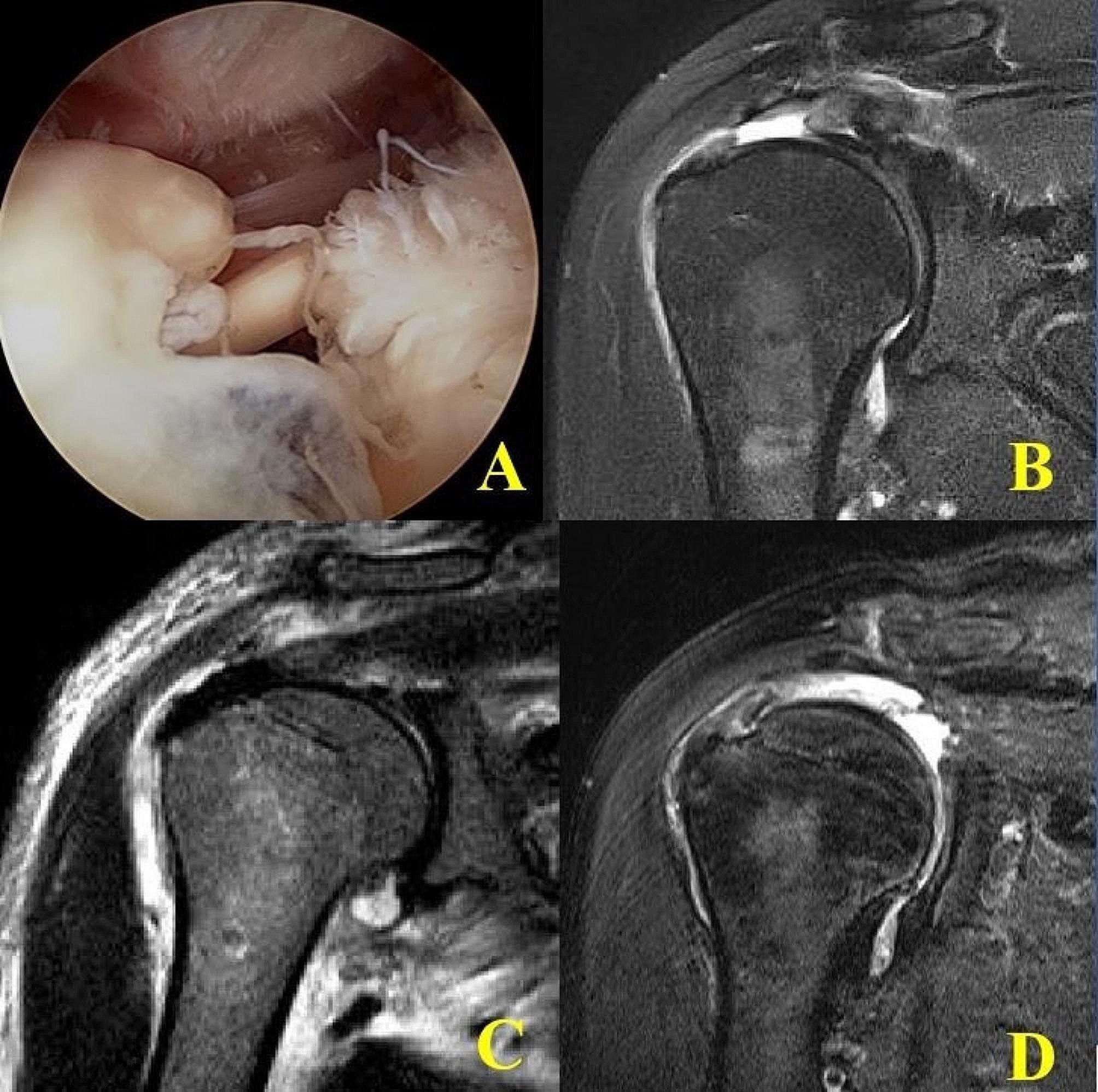
(**A**), (**B**) Arthroscopy and preoperative MRI showed that the cuff remnant on the footprint has a similar width to the original GT footprint and medially retracted tendon. (**C**) Immediate postoperative MRI showed good preservation of the remnant cuff on the greater tuberosity in the suture bridge rotator cuff repair. (**D**) 1-year of follow-up MRI showed large sized rotator cuff re-tear with remnant cuff (type 2 re-tear pattern)

127 patients included 71 medium size rotator cuff tears and 56 large size tears. In the remnant preserving group (Group I), the average preoperative tendon retraction from the most lateral border of the GT was 1.9 ± 0.9 cm. In the remnant debriding group (Group II), that was 2.1 ± 0.9 cm as measured on preoperative MRI. The degree of preoperative tendon retraction was not significantly different in both groups (AP: *p* = 0.288; ML: *p* = 0.148). In the remnant preserving group (Group I), there were 7 and 3 cases with fatty infiltration Grade 3 and 4, respectively. In the remnant debriding group (Group II), there were 6 cases each with fatty infiltration Grade 3 and 4. There was no statistically significant difference between the two groups (*p* = 0.599). The re-tear rate on MRI at 8 months after operation was 11/63 (17%) in group I (remnant preserving group) and 12/64 (19%) in group II (remnant debriding group) (Fig. 5). The difference in the re-tear rate (Sugaya type IV and V) was not statistically significant between the two groups (*p* = 0.053). The ratio of good-quality repaired tendons (Sugaya type I and II) and poor-quality repaired tendons (Sugaya type III, IV, and V) was statistically significantly higher in group I (*p* = 0.008) (Table 4).

**Table 4:** Comparison of Tendon Tear Size and Postoperative Tendon Quality Between Two Groups On MRI

	Group I (Remnant preserving) (*n* = 63)	Group II (Remnant debriding) (*n* = 64)	*p* value
Preoperative MRI			
Tear size, mm^2^	45.4	56.1	
Size, cm (AP)	2.2 ± 1.0	2.3 ± 1.0	0.288
Retraction, cm (ML)	1.9 ± 0.9	2.1 ± 0.9	0.148
SSP Fatty infiltration			
G0:G1:G2:G3:G4	9:23:21:7:3	19:22:14:6:6	0.599
Mean	1.54 ± 1.02	1.25 ± 1.12	
Postoperative MRI			
Sugaya classification type			
I, II(good quality) vs. III, IV, V(poor quality)	38:25	22:42	0.008
Re-tears, n (%)	11(17%)	12(19%)	0.053

NOTE. Data are presented as mean ± standard deviation unless otherwise indicated.

## Discussion

The most important finding of this study is that remnant preserving rotator cuff repairs yielded significantly improved quality of the repaired tendon as compared to remnant debriding repair. Remnant preserving rotator cuff repairs, facilitate tendon-to-tendon healing, are superior in terms of tendon quality and are the preferred option for transtendinous FTRCT. In a study by Sun et al. [13], which explored the impact of preserving remnant tendon tissue on tendon-to-bone healing biomechanically and histologically in a rabbit rotator cuff tear model, 26 New Zealand white rabbits underwent bilateral infraspinatus tenotomy. Repairs were performed using an open transosseous technique, preserving the remnant tendon on one side and removing it on the other. Biomechanical testing and histologic evaluation at 4 and 12 weeks revealed superior outcomes in the remnant tissue preservation group. These findings from Sun et al. support our most important finding.

Restoration of torn cuff tendon to its original anatomic footprint and complete coverage of footprint without tension are regarded as the most important factors to reduce re-tear rate of repaired rotator cuff [14–16]. On the other hand, whether repaired cuff integrity correlated with clinical outcomes is contentious. Some studies have reported that the structural integrity of a repaired cuff does not affect clinical outcomes [17–19], while others have reported that integrity of the repaired cuff is associated with clinical outcomes [18–20]. The latter studies found that fatty infiltration and muscle atrophy progresses after repair, with deteriorating clinical outcomes in long-term follow-up. Given this controversy, the best we can do to reduce re-tear rate or to preserve tendon integrity after rotator cuff repair is to make an anatomical restoration and complete coverage of footprint without tension. If the remnant cuff is debrided before repair, the tear size is likely to increase compared to remnant preserving cuff repair. Consequently, with the tear size proportionally influencing the tension of the suture, this could lead to an increase in the re-tear rate. Remnant preserving rotator cuff repair may be another way of reducing the uncertainty in the transtendinous FTRCT with remnant cuff.

Arthroscopic rotator cuff repair is a successful surgical method that provides significant pain relief and functional improvement of the shoulder joint in the majority of patients. An important step in rotator cuff repair is the tension-free anatomical reattachment of the tendon to the footprint of the GT. However, reattachment of the retracted torn tendon to the GT with the debridement of the remnant tendons in the transtendinous FTRCT with remnant cuff is not anatomical repair and can produce tension overload and tendon to bone healing. Rotator cuff repairs can be likened to constructing a suspension bridge designed to bear a distorted load. When the sharing of this load with nearby healthy tendons proves ineffective, it can lead to an elevated risk of re-tear or hindered tendon healing. Abnormal strains can result in issues with both cuff integrity and the range of motion (ROM) in the shoulder joint. Preventing over-loaded cuffs and similar tension with adjacent un-torn tendons is ideal [21–23]. Remnant preserving rotator cuff repair is a potential option for reducing tension overload in repaired tendon and avoid tension mismatch with adjacent tendons.

Conventionally, the primary goal of rotator cuff repair is considered tendon-to-bone healing [24]. However, rotator cuff repair healing after debridement of all remnant cuff is different from native tendon-to-bone healing. It is just scar-mediated healing. Angeline et al. [25] reported a scar-mediated healing response at the tendon-bone interface, which is notably weaker than the native enthesis and thus more prone to failure. The blood supply for the healing process is mainly from the bursal side. Gamradt et al. [26] reported that vascularity of the supraspinatus tendon 3 months after repair is from the peribursal tissue, followed by the anchor site, with the tendon remaining relatively avascular. Since the main sources of healing after cuff repair are peribursal tissue or anchor sites, rather than the GT footprint, there is no need to remove the remaining tendon to improve healing. Moreover, preservation of the remnant tendon tissues can be used to minimize mechanical mismatch and tension overload. We supposed that it might be enough to approximate both ends of the tendon (repaired tendon and remnant tendon) and form an interdigitation between tendon fibrils for tendon-to-tendon healing. Leung at al [27]. reported in a study on goats that tendon-to-tendon healing, rather than bone-to-tendon healing, is associated with better strength and failure load at 12 to 24 weeks. We supposed that tendon-to-tendon healing in the transition zone could produce a greater degree of durability and faster healing than tendon-to-bone healing.

Also, in view of the study of rotator cuff anatomy by Mochizuki et al. [6], it is possible that the transtendinous FTRCT with remnant cuff, if repaired by debriding the GT footprint, could be damaging intact infraspinatus insertion site as majority of GT is covered by infraspinatus according to this study. And on arthroscopy, it is not possible to clearly define the boundaries of supraspinatus and infraspinatus. So, surgeon might inadvertently remove the intact infraspinatus insertion site while debriding remnant cuff on the GT footprint. This anatomical detail should also be considered, and every attempt should be made to preserve remnant tendons on GT.

The concepts of remnant preservation of a torn cuff are not previously well described. Shin et al. [28] recently reported a retrospective study that rotator cuff repair with preservation of the remaining tendon on the footprint obtained satisfactory functional outcomes in several posterior L-shaped tear or transtendinous tear pattern with substantial remnant tendon. In addition, Walcott et al. [4] reported a case series that traumatic transtendinous FTRCT were repaired with an anatomically reduced, side-to-side technique. These reports are similar to our results about the effectiveness of remnant preserving repair. Furthermore, in remnant preserving procedure, we only decorticated a small portion of the GT for anchor insertion (next to the articular surface of the humeral head) to allow for tendon-to-tendon healing. And a posterolateral or lateral portal may not be sufficient to expose the anchor insertion site of the GT when anchor insertion using deadman angle. To prevent such difficulties, we preferred medial row anchor insertion through the Neviaser portal, rather than a lateral portal, using a 90º insertion angle and did no damage to the cuff remnants [29, 30]. For more preservation of cuff remnants on the GT footprint, multiple channeling and minimization of GT bone decortication and adequate anchor insertion through the Neviaser portal is a very useful technical tip.

Our study has several limitations. First, it had a retrospective design and a follow-up period (2-year follow-up) that could hinder proper evaluation of long-term clinical outcomes. Although the follow-up period was short, this study evaluated not only clinical outcomes but also re-tear rate and integrity of the repaired tendon through MRI. A second limitation is a selection bias. The duration from injury to surgery can have effect on cuff healing and functional outcomes. Acute rotator cuff tears are more likely to heal than are chronic rotator cuff tears. However, there was no statistically significant difference in the period between pain onset and surgery in the two groups. A third limitation is that our study included patients with advanced fatty infiltration (Grade 3 and 4) in both the groups, whether we preserved or debrided the remnant tendon. Typically, advanced fatty infiltration is considered a reason not to perform rotator cuff repairs and is excluded from inclusion criteria due to its potential impact on the healing process. However, in our investigation, we choose this inclusion based on the specific context of transtendinous FTRCT with a remnant cuff. The tear pattern and the presence of a robust remaining tendon played a role in shaping our decision. While our study provides valuable insights into comparing the two repair methods within this particular group of patients, it is essential to acknowledge that including cases with advanced fatty infiltration poses a limitation. For future studies, implementing stricter criteria for participant inclusion could enhance our understanding of outcomes by minimizing this potential complicating factor.

## Conclusions

Remnant preserving rotator cuff repairs demonstrated significantly better results in terms of the quality of the repaired tendon compared to remnant debriding repairs, despite no significant difference observed in re-tear rates. These results indicate that remnant preserving repairs favor superior tendon healing without compromising the native footprint, and the conventional remnant debriding cuff repair may be considered more conservatively in the transtendinous rotator cuff tears with remnant cuff.

### Electronic supplementary material

Below is the link to the electronic supplementary material.

Supplementary Material 1

Supplementary Material 2

## Data Availability

The datasets used and/or analyzed during the current study are available from the corresponding author on reasonable request.

## Abbreviations

GT: greater tuberosity
MRI: Magnetic resonance imaging
FTRCT: full thickness rotator cuff tear
UCLA: The University of California at Los Angeles
ASES: American Shoulder and Elbow Surgeons
SST: Simple Shoulder Test
CS: Constant Shoulder
ROM: range of motion
