# Supplementary material for: Arthroscopic cuff repair: footprint remnant preserving versus debriding rotator cuff repair of transtendinous rotator cuff tears with remnant cuff
Source: BMC Musculoskelet Disord. 2024 Apr 17;25:302. doi: 10.1186/s12891-024-07431-z (PMC11022446; doi:10.1186/s12891-024-07431-z)
Supplement: Supplementary file 2 — Supplementary Material 2 [file 12891_2024_7431_MOESM2_ESM.pdf]

Supplementary Material. Two-minute surgical video showing footprint remnant preserving rotator cuff repair of transtendinous rotator cuff tear with remnant cuff. Footprint Remnant Preserving Versus Debriding Rotator Cuff Repair of Transtendinous Rotator Cuff Tears with Remnant Cuff. After the intra-articular examination, in subacromial space, bursectomy and subacromial decompression were performed. After evaluation of the tear size, shape, and tendon mobility, the size of footprint remnants tendon was checked by using a calibrated probe. And then we made holes for medial anchors by Neviaser portal, anchor was inserted through the 90º angle. Medial row anchor insertion through “Neviaser portal” is very useful technical tips for remnants preservation. For the remnant preserving rotator cuff repair, a conventional suture bridge repair technique was used. All suture limbs of the medial row anchor were passed using a retrograde shuttle relay technique with a suture hook through retracted torn cuff but avoid the muscular portion of the torn retracted tendon. Medial-row repair was performed. These medial row tie set both tendon ends approximated and interdigitated to increase contact area for tendon-to-tendon healing. And then, knotless anchors for the lateral row repair were placed distal to the greater tuberosity with threads of the medial row anchors under tension to compress rotator cuff tendons across the GT footprint.
